# Targeting of CXCR3 improves anti-myeloma efficacy of adoptively transferred activated natural killer cells

**DOI:** 10.1186/s40425-019-0751-5

**Published:** 2019-11-07

**Authors:** Valentina Bonanni, Fabrizio Antonangeli, Angela Santoni, Giovanni Bernardini

**Affiliations:** 1 Department of Molecular Medicine, Sapienza University of Rome, Laboratory affiliated to Instituto Pasteur Italia-Fondazione Cenci Bolognetti, 00161 Rome, Italy; 2 IRCCS, Neuromed, Pozzilli, 86077 Isernia, Italy

**Keywords:** Multiple myeloma, CXCR3, Chemokines, Tumor immunotherapy, Cell migration

## Abstract

**Background:**

The peculiar multiple myeloma microenvironment, characterized by up-regulated levels of several inflammatory chemokines, including the CXCR3 receptor ligands CXCL9 and CXCL10, limits NK cell positioning into the bone marrow by interfering with CXCR4 function. It is still unclear if the consequent reduced influx of transferred cells into the tumor represents a potential limiting factor for the success of NK cell-based adoptive therapy. We hypothesize that inhibition of CXCR3 function on NK cells will result in increased tumor clearance, due to higher NK cell bone marrow infiltration.

**Methods:**

Since different activation protocols differently affect expression and function of homing receptors, we analyzed the bone marrow homing properties and anti-tumor efficacy of NK cells stimulated in vitro with two independent protocols. NK cells were purified from wild-type or *Cxcr3*^*−/−*^ mice and incubated with IL-15 alone or with a combination of IL-12, IL-15, IL-18 (IL-12/15/18). Alternatively, CXCR3 function was neutralized in vivo using a specific blocking antibody. NK cell functional behavior and tumor growth were analyzed in bone marrow samples by FACS analysis.

**Results:**

Both activation protocols promoted degranulation and IFN-γ production by donor NK cells infiltrating the bone marrow of tumor-bearing mice, although IL-15 promoted a faster but more transient acquisition of functional capacities. In addition, IL-15-activated cells accumulated more in the bone marrow in a short time but showed lower persistence in vivo. Targeting of CXCR3 increased the bone marrow homing capacity of IL-15 but not IL12/15/18 activated NK cells. This effect correlated with a superior and durable myeloma clearance capacity of transferred cells in vivo.

**Conclusions:**

Our results demonstrate that in vitro activation affects NK cell anti-myeloma activity in vivo by regulating their BM infiltration. Furthermore, we provided direct evidence that CXCR3 restrains NK cell anti-tumor capacity in vivo according to the activation protocol used, and that the effects of NK cell-based adoptive immunotherapy for multiple myeloma can be improved by increasing their bone marrow homing through CXCR3 inhibition.

## Background

Natural killer (NK) cells are cytotoxic innate lymphoid cells crucial for anti-tumor response and therefore attractive targets for the development of new cancer immunotherapies [1]. NK cells mainly differentiate from precursors in the bone marrow (BM) and following maturation leave the BM and migrate to peripheral tissues and lymphoid organs or reside in the BM as a reserve of effector cells for host defense [2, 3]. In steady state conditions, homeostatic chemoattractants contribute to NK cell release from BM into blood by promoting the migration of specific NK cell populations from BM parenchyma into the vasculature. During an immune response, BM mobilization of NK cells is enhanced by up-regulated expression levels of inflammatory chemokines that act both by inducing NK cell migration and by inhibiting CXCR4-mediated retention of NK cells into BM [3–9].

Tissue NK cells undergo a marked increase of effector capacity after an activation/priming phase promoted by cytokines, among which IL-15 is central [10]. In addition, NK cell populations with enhanced longevity features and robust response upon re-stimulation can be generated in vitro by activation with a combination of IL-12, IL-15, and IL-18 [11–14]. The possibility to produce a high number of highly cytotoxic effector NK cells with the capacity to persist in vivo has prompted the use of cytokines such as IL-2, IL-12, IL-15 and IL-18 to activate/expand in vitro human NK cells for adoptive immunotherapy [15–17]. Indeed, the use of IL-12/15/18-preactivated NK cells in combination with radiation therapy or with cytokine administration in vivo was shown to improve current protocols for immunotherapy of cancers in preclinical models and in clinical trials by sustaining their effector function and in vivo expansion [12, 18]. Nevertheless, it is still unclear whether distinct stimulation protocols can differently modulate NK cell anti-tumor function by influencing their tissue homing properties.

A key limitation for the use of NK cells in cancer therapy is the failure of activated/expanded adoptively transferred NK cells to traffic to the tumor site [16]. This may occur because the tumor microenvironment limits the recruitment of immune cells or because in vitro treatment promotes alteration of homing receptor expression [19, 20]. In this regard, significant advances in the promotion of NK cell tumor infiltration have been made thanks to enforced expression of chemoattractants in tumor cells or of chemokine receptors on NK cells, thus prolonging survival in several preclinical studies [21, 22]. Furthermore, it is still unclear if the choice of short-term in vitro activation as opposite to long-term expansion protocols can prevent the phenotypic changes on NK cells that are linked to tissue homing deficiencies and acquisition of an exhausted phenotype [16, 23, 24].

CXCR3 is a G protein-coupled receptor belonging to the family of chemokine receptors, expressed on several immune cell types including T helper, NKT, NK cells. CXCR3 is critical for NK cell infiltration into some solid tumor types upon IFN-γ-promoted induction of its ligands [21, 25]. On the other hand, activation of CXCR3 was associated with poor patient survival in several tumors due to its expression on tumor cells and on regulatory immune cell populations [26–28]. In addition, in the context of hematological tumors growing in BM, CXCR3 activation can lead to NK cell mobilization from BM into blood and thus may hinder NK cell accumulation in this anatomical compartment. CXCR3 ligands are up-regulated in multiple myeloma (MM), a plasma cell malignancy characterized by uncontrolled proliferation in BM, both in patients and in mouse models, and the up-regulation is associated with the severity of the disease and with poor patient survival [8, 17, 28]. In the mouse, CXCR3 ligand up-regulation occurs early during the asymptomatic phase of the disease and coincides with a decline of the host effector NK cell number in BM [8]. These observations support the prognostic role of CXCR3 ligands in MM but targeting of CXCR3/ligand axis for therapeutic purposes has not been investigated yet.

Considering the substantial ongoing effort to optimize NK cell-based immunotherapies supported by the promising anti-MM activity of expanded activated NK cells [29, 30], in this work we aimed to understand: i) if BM NK cell infiltration can be affected by changes of homing receptor expression and function occurring upon in vitro activation; ii) if inhibition of CXCR3 on NK cells could increase the efficacy of NK cell-based adoptive immunotherapy strategies. Thus, we activated mouse NK cells in vitro and monitored their functional status and changes of their migratory pattern in vitro and in vivo. Our findings suggest that reversal of CXCR3 inhibitory function on NK cell BM localization is a promising approach in MM therapy.

## Methods

### Mice and multiple myeloma (MM) mouse models

Female wild type (WT) Ly5.1 (CD45.1+) and Ly5.2 (CD45.2+) C57BL/6j mice (Charles River, Calco, Italy), Ly5.2 *Cxcr3*^*−/−*^*(*B6.129P2-Cxcr3tm1Dgen/J) mice (Jackson Laboratory, Bar Harbor, ME) were housed in the animal house of the Histology unit in Sapienza University and C57BL/KaLwRij mice (Envigo, Udine, Italy) were housed in the animal facility of Istituto Superiore di Sanità (Rome, Italy) under specific pathogen-free conditions.

All animal studies were designed according Animal Research: Reporting of In Vivo Experiments (ARRIVE) guidelines and to national (D.lgs. 26/2014) and international law and policies (EEC Council Directive 2010/63/EU) and were approved by the Italian Ministry of Health (Health Ministry authorization 769/2015 PR and 30/2015 PR).

Two million 5TGM1 or 1 × 10^6^ 5T33 MM cells (in 300 μL of PBS) were i.v. injected into six- to eight-week-old C57BL/KaLwRij mice. Mice were killed by cervical dislocation after 2 weeks (5T33) or after 3 and 4 weeks (5TGM1) for organ collection and analyses. Tumor burden was assessed at the time of mouse sacrifice by quantification of CD138^+^ cells in BM spleen and liver. IgG2b intracellular staining confirmed the frequency of monoclonal malignant plasma cells.

### Cell lines and reagents

5TGM1 and 5T33 multiple myeloma cell lines were kindly provided by Dr. Yoneda, (University of Texas, San Antonio, TX) and maintained in RPMI 1640 medium supplemented with 10% FBS, 2 mmol/L glutamine, 55 μmol/L β-mercaptoethanol and antibiotics. Cell lines were periodically authenticated by morphologic inspection, verified to be mycoplasma free, and were passaged for no more than 4 to 6 weeks from thawing.

Recombinant mouse IL-12, CXCL9, CXCL10 and human CXCL12, were from Peprotech EC (London, U.K.). Mouse IL-18 and IL-15 were from R&D systems. BSA, Carboxyfluorescein succinimidyl ester (CFSE), PKH26, Brefeldin A, Monensin and 7-Aminoactinomycin D (7-AAD) were from Sigma-Aldrich (St. Louis, MO, USA). Cytofix/Cytoperm TM Fixation/Permeabilization Kit was from BD Biosciences (San Diego, CA, USA).

### Primary NK cell activation

Spleens from naive C57BL/KaLwRij, C57BL/6 WT or *Cxcr3*^*−/−*^ mice were dissociated into single cell suspensions by mechanical disruption on a 70-μm cell strainer (Flacon, Becton Dickinson) with a rubber syringe plunger. NK cells were then enriched (80% purity) using NK Cell Isolation Kit (Miltenyi Biotech, Bergisch Gladbach, Germany). After 1 h recover in 37 °C 5%CO_2_ humidified incubator, purified NK cells were used or were activated by 20–22 h-cultures in complete Iscove’s Modified Eagle’s Medium (IMEM) supplemented with 100 ng/ml of IL-15 alone, with a combination of 10 ng/ml IL-12, 10 ng/ml IL-15, and 50 ng/ml IL-18 (IL12/15/18) or with low dose IL-15 (10 ng/ml to promote survival) [11]. Cells did not show significant size changes as assessed by Forward Scatter analysis. NK cells (CD3-NK1.1+) obtained from all culture conditions were more than 95% pure and were vital as assessed by propidium iodide staining and FACS analysis.

### Antibodies and flow cytometry analysis

mAbs directly conjugated to FITC, PE, PerCP 5.5, allophycocyanin, PEcyanine (cy)7, allophycocyanin-eFluor 780 and specific for the following antigens (clone name in parentheses) were used in this study: NK1.1 (PK136), CD3ε (145-2C11), CD45.2 (104), CD45.1 (A20), CD107a (ID4B), IFN-γ (XMG1.2), CD138 (281–2), IgG2b (RMG2b-1) CXCR3 (220,803 and CXCR3–173), CXCR4 (2B11), CD49d (R1–2), CD44 (IM7), GzmB (NGZB), Perforin (S16009B) and isotype controls were obtained from BD biosciences and from eBiosciences (Termo Fisher Scientific, Waltham, MA USA). Detection of intracellular mRNA encoding for CXCR4 was done by PrimeFlow RNA Assay using a type 1 probe according to the manufacturer’s instructions (Affymetrix and Thermo Fisher Scientific). All cells were analyzed by flow cytometry using a FACSCanto II (BD Biosciences), and data were elaborated using FlowJo Version 9.3.2 software (TreeStar).

### In vitro functional assays

Additional file 1 contains supplemental methods for degranulation, IFN-γ production and killing assays.

In vitro chemotaxis assays of activated NK cells in response to medium alone (NC), CXCL10 (250 ng/ml) and CXCL12 (200 ng/ml) were performed using 5-μm pore-size polycarbonate Transwell inserts (Sigma-Aldrich). The chemoattractants were diluted in migration medium (RPMI 0.5% BSA, 25 mM HEPES) and placed in the wells of the lower compartment. The cells (1 × 10^5^) were resuspended in migration medium and placed in the wells of the upper compartment. After incubation at 37 °C for 1 h, transwells were removed and the cells migrated across the filter were analyzed by flow cytometry as previously described [5].

The in vivo function of donor CFSE+ BM NK cells was determined immediately before transfer (day 0) and at 18 h and 48 h after in vivo transfer in MM-bearing mice by analyzing the CD107a (membrane) expression on freshly isolated cells and the IFN-γ (intracellular) expression by cells incubated for 4 h with Brefeldin A as described [7].

### Chemokine measurements

Additional file 1 contains supplemental methods for this section.

### Competitive adoptive transfer experiments

Previous experiments indicate that C57BL/6 and C57BL/KaLwRij donor NK cells have similar trafficking behaviour in a short time frame when transferred in C57BL/KaLwRij recipient mice [8]. Donor NK cells were isolated from the spleen of healthy C57BL/6 mice displaying CD45.1 variant and stimulated as described above. At the day of the experiments, NK cells were purified from spleen of C57BL/6 CD45.2 mice, mixed 1.1 with in vitro activated CD45.1+ NK cells and stained with the cell fluorescent dye CFSE (2.5 μM). CFSE+ NK cells (4 × 10^5^ cell/mouse) were i.v. transferred into tumor bearing mice 3 weeks after 5TGM1 cell injection. An aliquot was saved to calculate the input ratio. In some experiments, IL-15-stimulated WT (CD45.1) and *Cxcr3*^*−/−*^ (CD45.2) NK cells were mixed 1:1 and processed as above. At 18 h after transfer BM, spleen and peripheral blood cells were collected, and donor NK cells were identified according to their CFSE and CD45 allelic variant expression, their number was quantified and normalized according to the input cell number as previously described [7].

### Adoptive cell therapy

Purified NK cells from C57BL/KaLwRij mice were activated in vitro, stained with CFSE and 5 × 10^5^ cells were i.v. transferred into mice 3 weeks after tumor injection. To define the role of *Cxcr3* deficiency in NK cell-mediated anti-MM effect in vivo, donor NK cells were purified from spleen of C57BL/6j WT and *Cxcr3*^*−/−*^ mice, stimulated in vitro and transferred as above. Our experiments demonstrated that IL-15-activated donor NK cells from C57BL/6j versus C57BL/KaLwRij mice had similar in vivo inhibitory effects on tumor growth in a 48-h time frame (not shown). Control (NT) tumor-bearing mice were i.v. injected with PBS. Tumor burden was assessed after mice sacrifice at 48 h after transfer. For long-term studies, recombinant IL-15 (50 μg/Kg) was administered i.v. to mice 18 h after NK cell transfer and tumor burden was analyzed 6–7 days thereafter. CXCR3 blocking in vivo was performed using the mAb clone CXCR3–173 from Bio-X-cell (West Lebanon, NH, USA). CXCR3–173 mAb or control hamster IgG (250 μg/mouse) were i.v. administered at day − 1 and + 1 of NK cell transfer.

### Statistics

Sample size was defined on the basis of past experience on the MM models, in order to detect differences of 20% or greater between the groups. Values were expressed as mean ± standard error of mean (SEM) of biological replicates, as specified. One-way ANOVA or unpaired (or paired to analyze competitive adoptive transfer experiments) student’s t test were used to compare multiple groups. A *p* ≤ 0.05 was considered statistically significant. Statistics were calculated with GraphPad Prism version 6, GraphPad Software.

## Results

### In vitro activated NK cells reduce MM burden upon adoptive transfer depending on the type of stimuli

In order to identify an in vitro activation protocol that could be effectively used in NK cell based adoptive cell therapy of MM, we assessed the anti-myeloma efficacy in vivo of NK cells activated by two protocols currently used in preclinical models and in clinical trials [12, 18, 30]. Purified NK cells were activated for 20–22 h with IL-15 alone, or with a combination of IL-12, IL-15 and IL-18 (IL-12/15/18) and stained with CFSE.

**Fig. 1:**
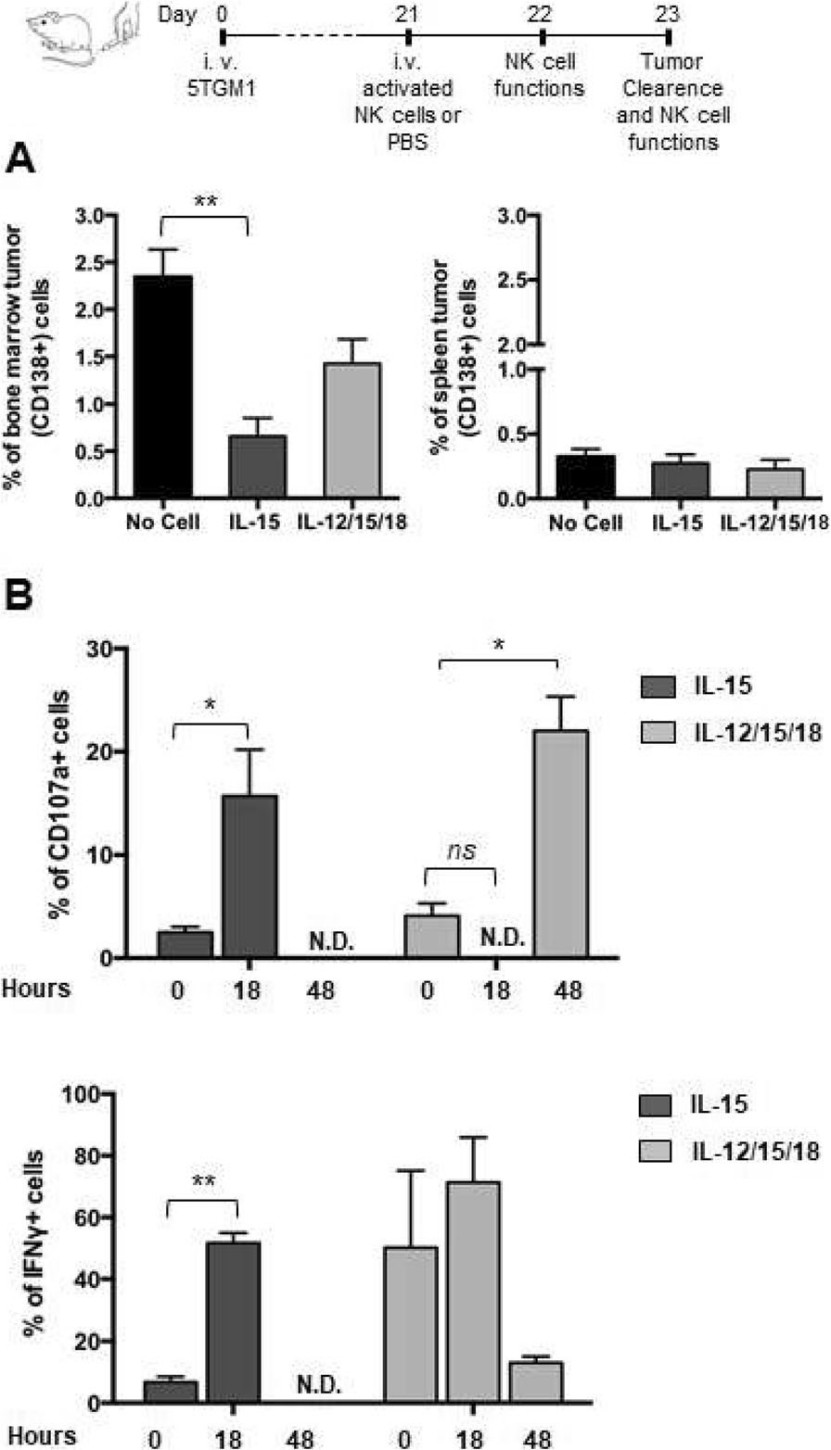
Anti-MM efficacy and in vivo functional status of activated NK cells. Activated (5 × 10^5^) CFSE+ NK cells obtained from splenocytes of C57BL/KaLwRij or PBS (No Cell) were i.v. transferred into MM-bearing mice 3 weeks after 5TGM1 cell injection. **a**) Tumor growth was determined by FACS analysis of CD138+ (tumor) cells among BM (2 tibias and femurs) and spleen cells at 48 h after transfer. The average ± SEM of 3 independent experiments with a total of at least 8 animals per group is shown. **b**) Activated NK cell functions in BM were determined by FACS analysis of CD107a + and IFN-γ + donor cell frequency 18 h and 48 h after transfer in MM-bearing mice. Graphs show average frequency ± SEM of CD107a + and IFN-γ + donor cells from 2 independent experiments, *n* = 5 per group. Time 0 corresponds to NK cell function immediately before the transfer. ND: Not detectable. Student t test was performed to compare no cell versus activated NK cell transferred mice (**a**) or differences between time 0 versus 18 h or 48 h (**b**). *p < 0.05; **p < 0.01

Transfer of IL-15-stimulated cells reduced substantially (60–70% decrease of CD138+ tumor cell frequency) tumor cell burden in BM as compared to PBS-injected mice, while IL-12/15/18 activated cells were less effective (Fig. 1a). Tumor cell reduction was confirmed by intracellular analysis of IgG2b expression, a monoclonal protein marker of MM cells (Additional file 2: Fig. S1A).

To correlate the anti-tumor effects with NK cell functional status in vivo, we monitored CD107a and IFN-γ expression by donor (CFSE+) BM NK cells in tumor-bearing mice (Fig. 1b). When compared to the cells before the transfer, the frequency of CD107a^+^ and IFN-γ^+^ IL-15-activated NK cells increased at 18 h and declined to zero at 48 h after transfer. The frequency of IFN-γ^+^ IL12/15/18-stimulated NK cells was already elevated before transfer into tumor-bearing mice, persisted at similar levels at 18 h and declined after 48 h, when the frequency of CD107a + cells had increased.

A greater acquisition of degranulation capacity by IL-15 activation compared to IL-12/15/18-activation was also demonstrated in vitro, as determined by analysis of membrane CD107a^+^ NK cell frequency upon incubation with 5TGM-1 cells or stimulation with anti-NKG2D mAb (Additional file 2: Fig. S1B). Membrane expression of NKG2D and killing of 5TGM1 cells were similar (Additional file 2: Fig. S1C). On the other hand, IL-12/15/18-activated NK cells produced more IFN-γ than IL-15-activated NK cells regardless of NKG2D triggering due to the synergistic effect of IL-12 with IL-18 [11] (Additional file 2: Fig. S1D).

### NK cell tissue migration in vivo is modulated by the mode of cytokine activation

In addition to the activation of effector functions, an important component of NK cell anti-tumor response is the ability to accumulate at the tumor site. We previously documented that NK cell migration to BM is impaired in MM-bearing mice [8]. Thus, we were interested to understand if the type of activation protocol could change the negative effect of the tumor microenvironment on BM NK cell homing and if this could be associated with a better anti-tumor response.

**Fig. 2:**
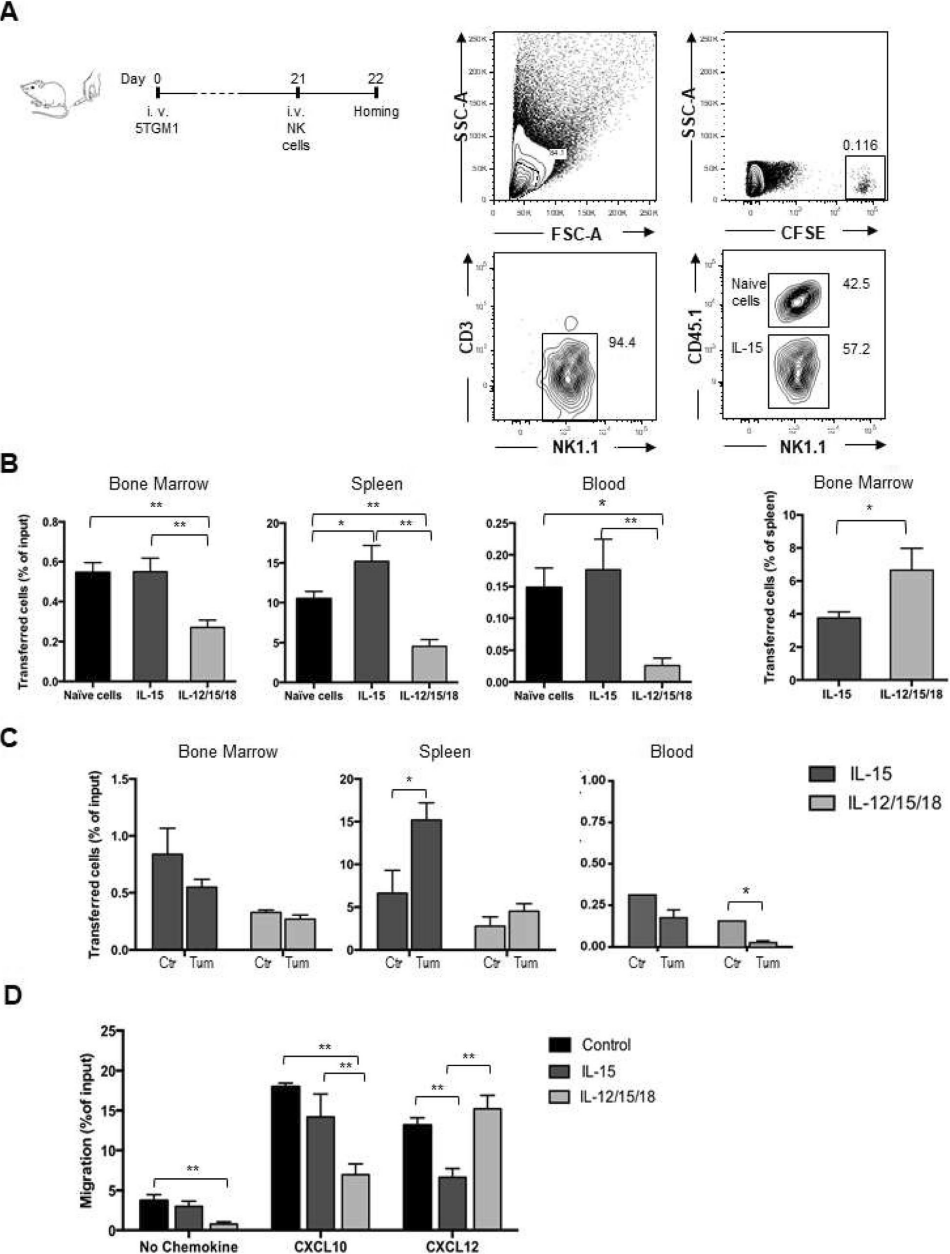
In vivo tissue migration and in vitro chemotaxis of activated NK cells. Activated CD45.1+ NK cells were mixed 1:1 with freshly isolated (naïve) CD45.2 NK cells, stained with CFSE and i.v. transferred into C57BL/KaLwRij mice 3 weeks after tumor cell injection. NK cell number was determined after 18 h in BM (two tibias and femurs), spleen and blood by FACS analysis of CD45.1+ or CD45.2+ NK1.1+ cells within donor CFSE+ cells and normalized with the number of input cells (% of input cells). **a**) Dot plots show the gating strategy for the analysis of IL-15 activated donor NK cells in the spleen of MM-bearing mice. **b**) CFSE+ cells were enumerated in each organ and frequency of donor cells out of transferred (input) cells is shown as average ± SEM of 2 independent experiments *n* = 5 mice per groups. Right hand graph: BM homing of activated donor NK cells was normalized by donor cell frequency into spleen. **c**) Tissue migration of activated NK cells in healthy control (ctrl) and tumor-bearing mice (tum). One-way ANOVA with multiple comparison was performed to compare tissue distribution of activated cells and naïve cells (**b**) and of activated cells in ctr and tum (**c**). *p < 0.05; **p < 0.01 **d**) In vitro chemotaxis assay of activated or control (cells treated with IL-15 10 ng/ml) NK cells in response to medium alone (no chemokine), to CXCL10 (250 ng/ml) or to CXCL12 (200 ng/ml). Results show average ± SEM from 3 independent experiments. One-way ANOVA was performed to compare migration of activated cells versus control cells. *p < 0.05; **p < 0.01

To perform competitive adoptive transfer experiments in MM-bearing mice, freshly isolated (naïve) donor NK cells displaying the CD45.2 variant were mixed 1:1 with activated CD45.1+ NK cells and i.v injected in MM-bearing mice (Fig. 2a).

As shown in Fig. 2b, 18 h after transfer the infiltration of donor IL-12/15/18-stimulated NK cells in all tissue compartments investigated was lower than IL-15-activated NK cells. We attributed this numeric reduction to a greater need for cytokine re-stimulation by IL-12/15/18 activated NK cells than IL-15 activated cells to survive in vivo [12, 14, 31, 32]. To compare the BM-homing ability of NK cells stimulated in different conditions with respect to migration to peripheral organs, we normalized their number in BM with that of the spleen, demonstrating that IL-12/15/18-stimulated cells were more prone to home to the BM than the IL-15-activated counterpart (Fig. 2b right panel). Naïve and IL-15 activated NK cell BM infiltration were comparable.

In vivo migration of IL-15 activated NK cells was affected by the tumor as we observed a slight reduction of transferred NK cell homing capacity in the BM and a 2.5-fold enhancement of splenic infiltration in MM-bearing compared to healthy control mice (Fig. 2c).

In order to correlate changes in BM homing with the function and expression of receptors important for NK cell trafficking to BM, we assessed the in vitro chemotaxis of purified NK cells in response to CXCL10, the ligand for CXCR3, and to CXCL12, the ligand for CXCR4 after incubation with IL-15 alone, with the IL-12/15/18 combination, with low-dose IL-15 alone (control: 10 ng/ml, used as a survival factor) or freshly isolated (naïve). NK cells activated with IL-12/15/18 migrated less than IL-15 activated and control cells in response to CXCL10, while IL-15 activated cells migrated less to CXCL12 (Fig. 2d). Of interest, migration of IL-12/15/18 NK cells was impaired even in the absence of chemokines, suggesting that these cells have decreased motility or that they express factors that counteract their migration to the lower chamber (Fig. 2d).

**Fig. 3:**
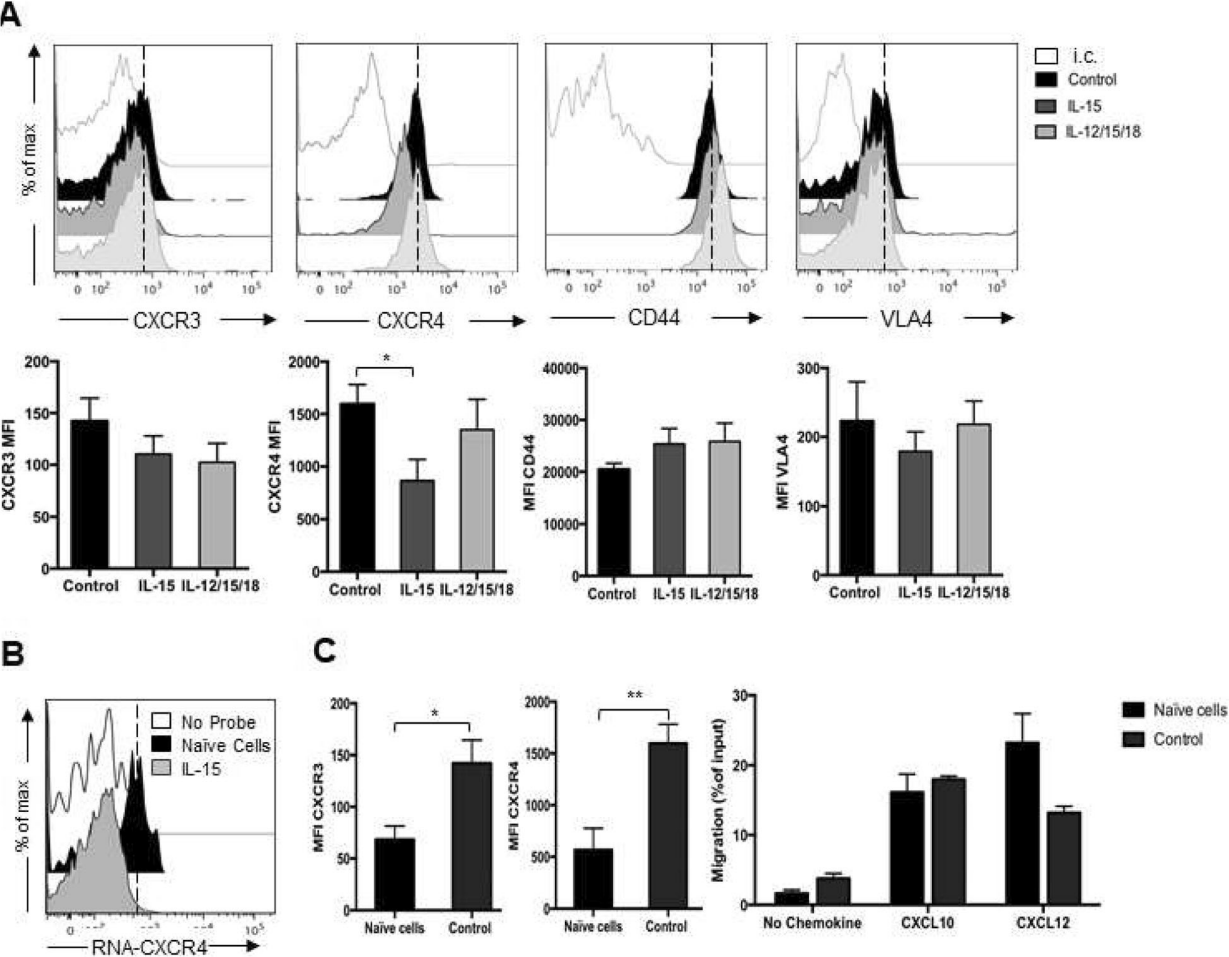
Expression of homing receptors on activated NK cells and NK cell migration in vitro**.** Purified NK cells were activated with IL-15, IL-12/15/18 for 20 h (control cells: IL-15 10 ng/ml). NK cell purity was assessed by anti-NK1.1 and -CD3 staining and expression of CXCR3, CXCR4, CD44 and CD49d (VLA-4) integrin chain was determined using specific antibodies. **a**) Upper panels show histogram plot of overlays of receptor staining in untreated and cytokine treated cells of a representative analysis. White filled histograms represent isotype control (i.c.) staining. Lower panels show average ± SEM median fluorescence intensity (MFI) from at least 3 independent analysis. Non-specific staining was subtracted from analysis. **b**) Detection of intracellular mRNA encoding for CXCR4 was done by PrimeFlow RNA Assay. **c**) Comparison between naïve and control cell receptor expression and migration: Left graphs show average ± SEM median fluorescence intensity (MFI) of CXCR3 and CXCR4 receptor. Right, in vitro chemotaxis assay in response to medium alone (no chemokine), to CXCL10 (250 ng/ml) or CXCL12 (200 ng/ml). Results show average ± SEM from 2 independent experiments

Compared to IL-12/15/18 activated and control cells, IL-15 stimulation did not significantly modulate the membrane expression levels of CXCR3, while CXCR4 membrane expression levels were reduced (Fig. 3a). Inhibition of CXCR4 expression by IL-15 occurred at the mRNA level as determined by intracellular flow cytometry analysis (Fig. 3b). Compared to naïve cells, control cells had increased membrane expression levels of both CXCR3 and CXCR4 compared to freshly isolated cells, but migration to their respective ligands was similar (Fig. 3c). None of the activation protocols significantly modified the membrane expression levels of other key receptors for leukocyte homing to BM, namely the cell surface adhesion receptors CD44 and very late antigen (VLA)-4 [33, 34].

Overall, these experiments indicate that IL-15 activated NK cells have reduced trafficking capacity to the BM compared to IL-12/15/18 cells, this correlating with lower CXCR4 expression levels and function, but display a higher frequency in circulation after adoptive transfer and have thus a better BM infiltration.

### *Cxcr3* deficiency enhances activated NK cell homing to BM and clearance of established tumors

Since the administration of NK cells activated with each protocol results in tumor regression over a short time frame but is associated with little BM NK cell infiltration, we attempted to potentiate anti-tumor efficacy by unleashing their BM tropism. Up-regulation of CXCR3 ligands hampers CXCR3^+^ NK cell migration into BM of MM-bearing mice [8]. The relevance of this effect for immunotherapy based on the adoptive transfer of activated NK cells has not been investigated yet.

**Fig. 4:**
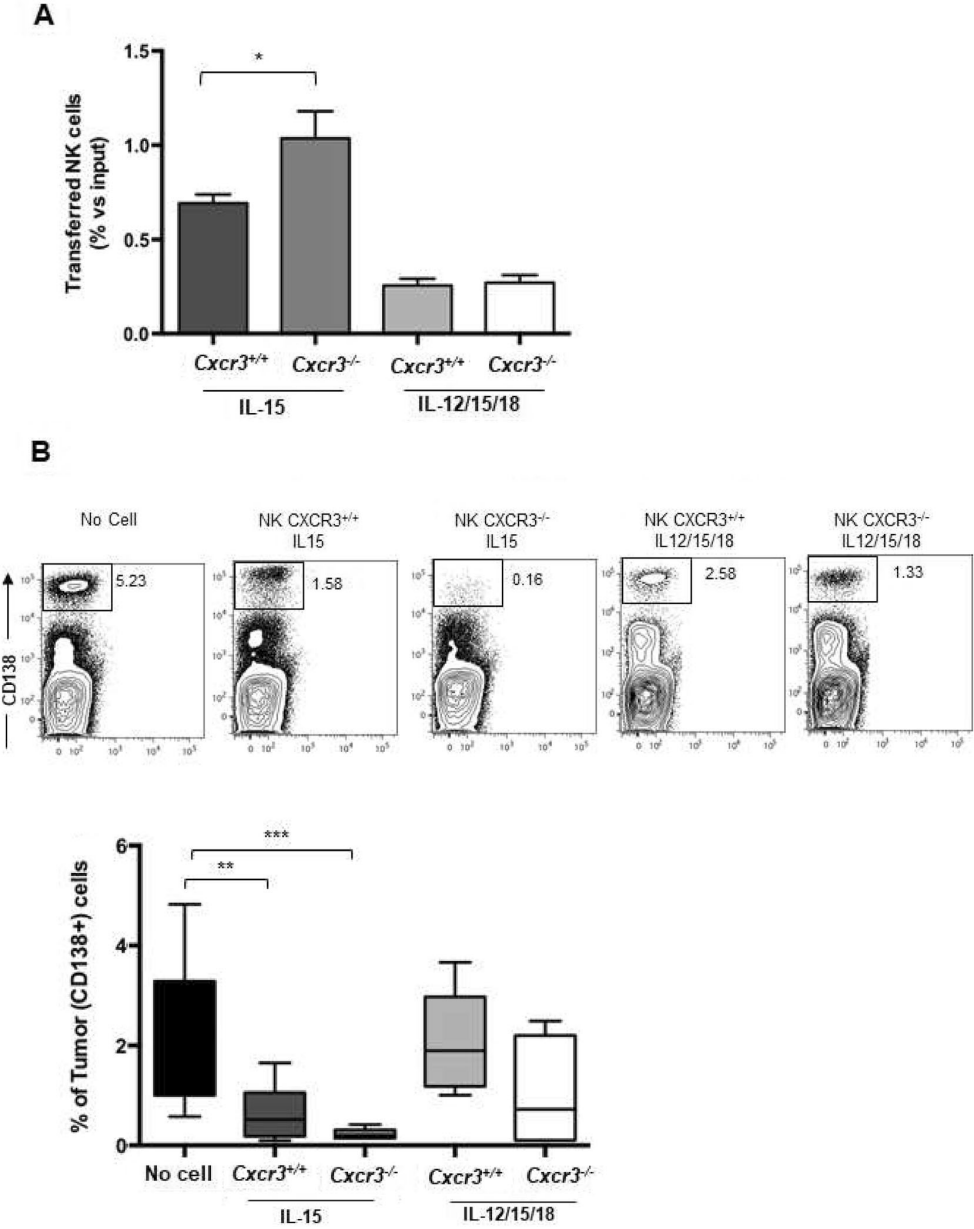
In vivo tissue distribution and anti-MM efficacy of IL-15 activated WT versus *Cxcr3* deficient NK cells. **a**) Activated CFSE+ NK cells (4 × 10^5^) composed of *Cxcr3*^*+/+*^ (CD45.1+) and *Cxcr3*^*−/−*^ (CD45.2+) cells mixed 1:1 were transferred to tumor-bearing mice and donor cell number into tissues was quantified and normalized on input cells after 18 h. The number of transferred (donor) cells is shown in panel A as average ± SEM of frequency of input cell number. Two independent experiments with a total of at least 5 recipient mice per group were performed. **b**) Activated NK cells (5 × 10^5^) from *Cxcr3*^*+/+*^ or *Cxcr3*^*−/−*^ mice were transferred to MM-bearing mice and tumor burden was calculated after 48 h. Upper panel shows a representative analysis of frequency of CD138+ cells in the different conditions tested. Lower panel shows average ± SEM of frequency of tumor cells in BM from two independent experiments using a total of at least 6 animals per group. One-way ANOVA test was used to compare multiple groups. *p < 0.05;**p < 0.01; ***p < 0.005

By performing competitive homing experiments in tumor-bearing mice, we demonstrated that *Cxcr3*^*−/−*^ deficiency promoted increased BM infiltration for IL-15 activated but not IL12/15/18 activated *Cxcr3*^*−/−*^ NK cells compared to the wild-type counterparts (Fig. 4a). This was not associated with differences of CXCR4 membrane expression levels on *Cxcr3*^*−/−*^ vs *Cxcr3*^*+/+*^ activated NK cells (Additional file 3: Fig. S2). More importantly, higher BM infiltration by *Cxcr3*^*−/−*^ NK cells inversely correlated with tumor burden. Tumor cell frequency was reduced by 60% after the transfer of IL-15-activated *Cxcr3*^*+/+*^ NK cells while it was reduced by more than 85% after the transfer of the *Cxcr3*^*−/−*^ counterpart when compared to PBS-injected mice. On the other hand, *Cxcr3* deficiency had minimal effects on IL-12/15/18-activated NK cells anti-tumor activity in vivo (Fig. 4b). The effect of *Cxcr3* deficiency on NK cell-mediated MM clearance was confirmed using the 5 T33 MM model (Additional file 4: Fig. S3A). Upon transfer of IL-15 activated *Cxcr3*^*−/−*^ NK cells, 5T33-bearing mice showed more than 50% reduction of tumor cell frequency in BM compared with mice treated with *Cxcr3*^*+/+*^ IL-15 activated cells or vehicle. *Cxcr3* targeting did not affect tumor growth in the spleen in the 5TGM1 and 5 T33 MM models, which is low due to NK cell surveillance [8] (Additional file 4: Fig. S3A and B).

### IL-12/15/18 but not IL-15 activated NK cells promote long-term tumor regression after IL-15 re-stimulation in vivo

Since IL-12/15/18 activated NK cells appear to have a numerical and functional disadvantage in a short time frame, while the activation of IL-15-activated NK cell functions in vivo is transient, we analysed longer time points to better assess the therapeutic outcome of their adoptive transfer. Since we could not observe any significant difference of tumor growth between PBS and activated NK cell-treated mice at 1 week after transfer (data not shown), we hypothesized a defective persistence of transferred cells in vivo. Thus, mice were administered with IL-15 to prolong donor NK cell survival [32]. Activated NK cells were i.v. transferred into mice 3 weeks after MM cell injection and tumor growth was determined 7 days later.

**Fig. 5:**
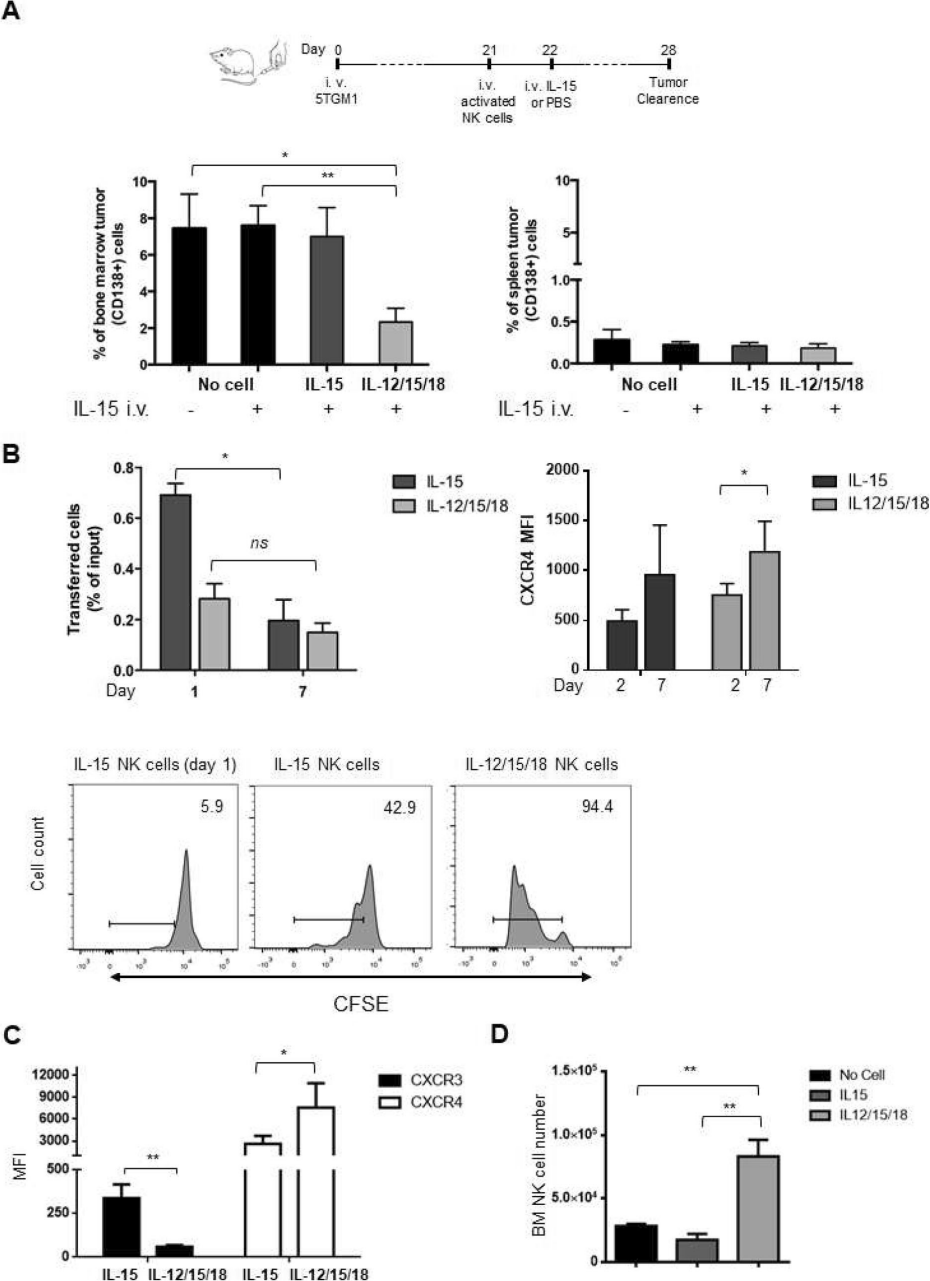
Long term anti-MM efficacy of activated NK cells. Activated NK cells (5-6 × 10^5^), obtained from splenocytes of C57BL/KaLwRij, or PBS (No Cell) were i.v. transferred into MM-bearing mice 3 weeks after 5TGM1 cell injection and IL-15 was administered 18 h later. **a**) Average ± SEM of frequency of tumor cells in BM and spleen 7 days after adoptive transfer (n = 5 in two independent experiments). One-way ANOVA test was used to compare multiple groups. **b**) BM persistence of transferred activated NK cells: IL-15 or IL-12/15/18 activated CFSE+ NK cells (4 × 10^5^) were transferred to tumor-bearing mice and donor cells were enumerated in BM and spleen 1 and 7 days later. Upper left graph shows the number of donor cells as average ± SEM of frequency of input (transferred) cell number. Student *t* test was performed to analyze the difference between day 1 and day 7. Upper right graph shows average MFI ± SD of CXCR4 expression levels on donor NK cells 2 and 7 days after transfer (*n* = 3 per group; one experiment). Lower panels, 7 days after transfer, in vivo proliferation of transferred NK cells was analyzed by CFSE dilution. Histograms were gated on NK1.1+ transferred NK cells and one representative histogram from each group is shown (n = 3 per group). Numbers in the histogram plot indicate the percentage of cells that had proliferated. **c**) Average MFI ± SEM of CXCR3 and CXCR4 expression levels on activated NK cells cultured in vitro for 7 days in low IL-15 concentration (10 ng/ml). **d**) Endogenous NK cell number was determined in BM (two tibias and femurs) by FACS analysis of CD3-NK1.1+ cells in the CFSE^−^ population.*p < 0.05;**p < 0.01

As shown in Fig. 5a, only IL-12/15/18 activated NK cells displayed a marked capacity to reduce tumor burden in BM compared to control (no cell) mice. Indeed, IL-12/15/18-activated NK cells were able to decrease tumor cell frequency in BM by 80%. On the contrary, IL-15 treatment alone or in combination with IL-15 activated NK cell transfer was ineffective. This was associated with lower BM persistence of IL-15 activated cells since their BM infiltration drastically dropped 7 days after transfer, while IL-12/15/18-activated NK cell number remained stable (Fig. 5b, upper left panel). The longer persistence of BM IL-12/15/18-activated NK cell was associated with a higher proliferation rate in vivo compared to IL-15 activated cells (Fig. 5b lower panels). In addition, in vivo expression of CXCR4 on IL-12/15/18 transferred cells in BM was significantly up-regulated at day 7 compared to day 2 and was slightly higher than IL-15 activated cells and was not significantly regulated by IL15 administration (Fig. 5b, upper right and Additional file 5: Fig. S4 A and B). Thus, to determine if longer persistence of IL-12/15/18-activated NK cells could be associated with regulation of chemokine receptor expression levels due to cytokine stimulation, we performed long-term in vitro cultures of activated NK cells. After 7 days, IL-12/15/18-activated NK cells displayed higher CXCR4 expression levels than IL-15 activated cells and almost completely loose the expression of CXCR3 (Fig. 4c and Additional file 5: Fig. S4C). Finally, the superior anti-tumor efficacy of IL-12/15/18-activated NK cells was associated with higher number of endogenous NK cells, an effect that was not observed at day 2 post-transfer (Fig. 4d and Additional file 6: Fig. S5A). This drastic change of NK cell number was not attributable to changes of CXCR4 and CXCR3 ligand expression levels since CXCL10 expression in the BM was not modulated by NK cell transfer or IL-15 administration, nor was the expression of CXCL10 and CXCL12 by BM tumor cells (Additional file 6: Fig. S5B).

### CXCR3 blockade induces long-term anti-tumor effect of IL-15-activated NK cells in vivo

Since the reduced NK cell localization and anti-tumor efficacy in MM can be reverted by CXCR3 deficiency in the short time frame (48 h), we wanted to demonstrate that CXCR3/ligand axes can be exploited in NK cell-based immunotherapy by showing a long-term effect of CXCR3 inhibition. Thus, we inhibited CXCR3 function by in vivo administration of an anti-CXCR3 blocking monoclonal antibody (CXCR3–173). CXCR3–173 mAb or control hamster IgG were given i.v. 1 day before and 1 day after the intravenous administration of activated NK cells.

**Fig. 6:**
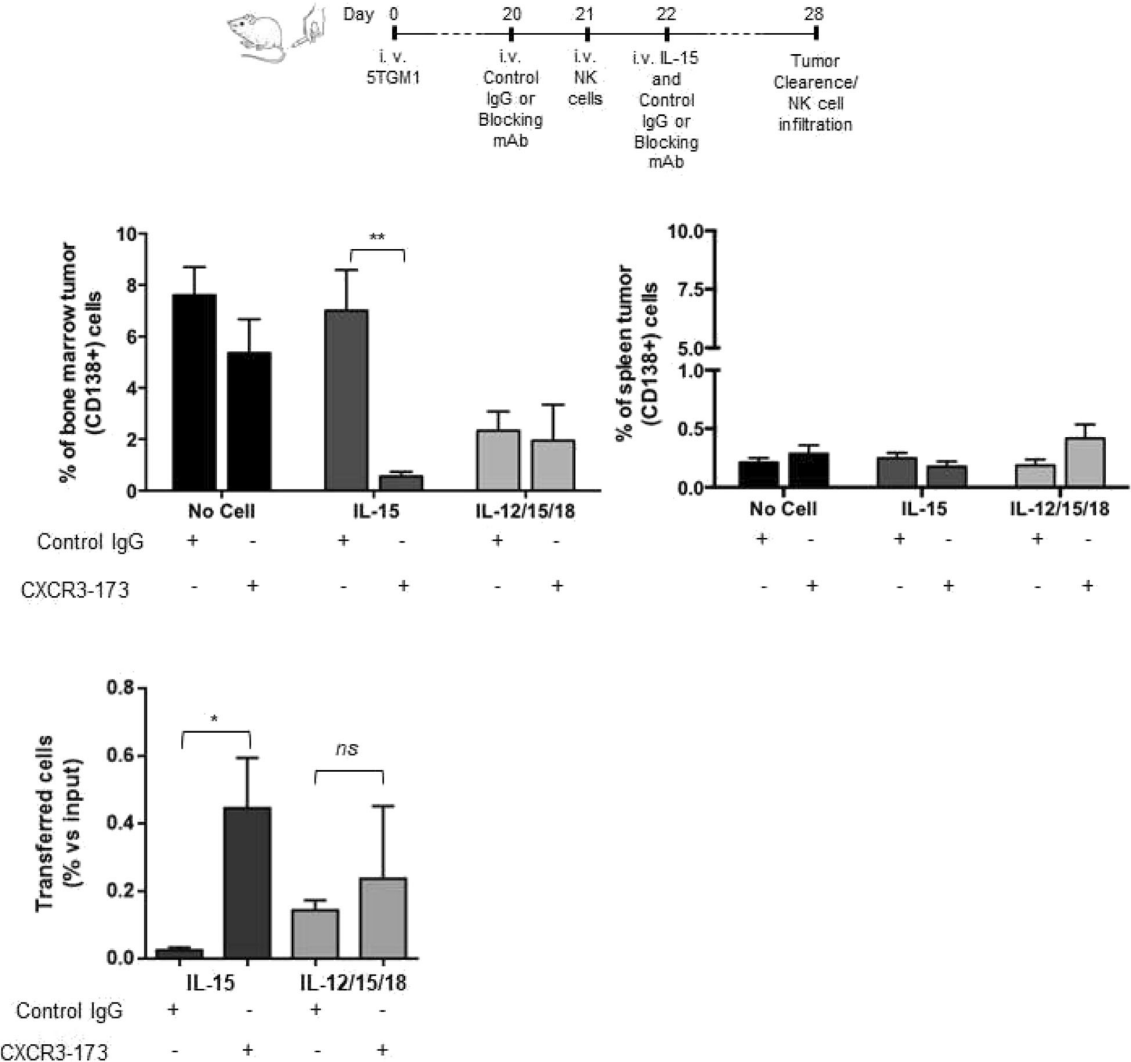
Long term anti-MM efficacy of IL-15 activated NK cells after CXCR3 blockade. Activated NK cells (6 × 10^5^) were i.v. transferred into MM-bearing mice 3 weeks after 5TGM1 cell injection with control hamster IgG or anti-CXCR3 blocking mAb (2 doses of 250 μg). Tumor growth was determined by FACS analysis of CD138+ (tumor) cells among BM cells 7 days after transfer. Lower graph: Activated NK cells were labeled with 2.5 μM CFSE and adoptively transferred in tumor-bearing mice and donor BM NK cell number was determined 7 days later by FACS analysis of CD3-NK1.1+ cells within donor CFSE+ cells and normalized with the number of input cells (% of input cells). Average number ± SEM of 2 independent experiments (*n* = 4 mice/group) is shown. Student t test was performed to compare control Ig versus anti-CXCR3 treated mice. **p < 0.01

As shown in Fig. 6, anti-CXCR3 mAb treatment in combination with IL-15-activated NK cells and recombinant IL-15 markedly reduced tumor cell burden in the BM while the combination with control IgG had no protective effect. Conversely, the efficacy of IL-12/15/18-activated NK cells remained unchanged in anti-CXCR3 mAb-treated compared to control IgG-treated mice. The therapeutic effect of CXCR3–173 mAb was not mediated by host cells or by inhibition of CXCR3 on tumor cells since the administration of CXCR3–173 mAb without adoptive cell transfer did not show protective effects. Moreover, we demonstrated that the prolonged efficacy of IL-15 activated cells was associated with the effect of antibody administration on NK cell BM infiltration. Indeed, NK cells displayed a 2- to 3-fold increase of BM accumulation in MM-bearing mice 18 h after in vivo administration of CXCR3–173 mAb (Additional file 4: Fig. S3C). This corresponded to increased accumulation of IL-15 but not IL-12/15/18-activated cells in BM upon antibody treatment (Fig. 6, lower panel).

## Discussion

Despite promising findings showing NK cell efficacy against MM, strategies attempting to overcome the impairment of NK cell tumor infiltration to improve their therapeutic effect are still in their infancy. Our results demonstrate that: i) in the BM of tumor-bearing mice, IL-15-activated NK cells have a numerical and functional advantage in the short time-frame compared to the IL-12/15/18 counterpart, allowing a superior tumor clearance capacity; ii) in vivo re-stimulation of donor NK cells with a single dose of IL-15 promotes long-term anti-tumor effect of NK cells pre-activated with IL-12/15/18; iii) inhibition of CXCR3/ligand axes potentiates the ability to infiltrate BM and enhances anti-MM activity of IL-15 activated NK cell.

Since the overall goal of this study was to determine if strategies that counteract the inhibition of NK cell homing to BM can improve NK cell efficacy against established MM, we initially chose a short time frame (48 h) to evaluate tumor burden in relation to the migration and functional capacity of adoptively transferred activated syngeneic NK cells. Afterwards, we analyzed the persistence of NK cell anti-tumor effect by determining tumor burden 7 days after adoptive transfer.

Our analyses indicate that in vitro activated NK cells display anti-tumor capacity when transferred in MM-bearing mice that depends on their functional status and on the ability to infiltrate the BM. IL-15-stimulated NK cells showed a faster degranulation response in vivo than IL-12/15/18 that may account for their higher capacity to restrain tumor burden within a short time-frame (48 h). The slower degranulation kinetics of IL-12/15/18 activated NK cells in vivo corresponded to lower degranulation capacity in vitro in response to stimulation with MM tumor cells or upon triggering of NKG2D, a receptor critically involved in NK cell response to MM [35, 36]. Nevertheless, the killing capacity of activated NK cells was similar, suggesting that IL-12/15/18 activation generates more efficient killer cells.

NK cell functions and migration are controlled by different chemokines and NK cell stimulation with cytokines can lead to changes in the expression of chemokine receptors [24, 37]. Of interest for this study, NK cell homing and retention in BM is controlled by several homing receptors, and alteration of CXCR4/CXCL12 and CXCR3/ligand axes was shown to correlate with the decrease of effector NK cell distribution and function in the BM of MM patients and of MM bearing mice [8, 38]. IL-15 but not IL-12/15/18 activation decreased CXCR4 expression and the chemotactic response to CXCL12 in vitro. This result correlates with higher BM homing capacity of IL12/15/18 compared to IL-15 activated NK cells, although the BM accumulation of the former was much lower due to the reduction of their number in vivo compared to IL-15 activated cells.

Both IL-15 and IL-12/15/18 activated NK cell anti-tumor effects in vivo were transient when these cells were used alone, supporting the importance of optimizing NK cell infiltration and/or persistence at the tumor site to reach high number of effector cells able to rapidly kill tumor cells.

Our data demonstrate for the first time that it is possible to increase activated BM NK cell infiltration with beneficial anti-myeloma effects by genetic deletion of *Cxcr3* gene or by in vivo administration of anti-CXCR3 specific mAb. These results correlate with the negative role of CXCR3 activation on BM NK cell localization and with up-regulated levels of CXCR3 ligands in the MM-microenvironment [8, 39, 40].

Miller and co-workers previously demonstrated that persistence of transferred human NK cells in host tissues is prolonged by in vivo cytokine administration [41]. Thus, we prolonged activated NK cell persistence in vivo by i.v. administration of recombinant IL-15 and analyzed differences in the two activation protocols after 7 days. IL-12/15/18- but not IL-15-activated NK cells were markedly effective in restraining tumor growth as determined by analysis of tumor burden 7 days after adoptive cell transfer, this correlating with their higher BM persistence and proliferation rate. The beneficial action of IL-15 in vivo administration was mediated exclusively by the transferred cells and not by the host cells since there was no decrease in the tumor burden in mice administered only with IL-15, consistent with what demonstrated previously [42]. Moreover, we demonstrated that the inhibition of CXCR3/CXCL10 axis can be exploited in NK cell-based immunotherapy by mAb-based neutralization of CXCR3 in vivo. Our data indicate that CXCR3 blockade induces a powerful long-lasting anti-tumor effect due to the potentiation of their accumulation in BM. Of relevance, the combination of mAb treatment and NK cell infusion allowed a more pronounced effect when using IL-15 compared to the IL-12/15/18 activated NK cells. Indeed, we observed no improvement in the efficacy of IL-12/15/18-stimulated NK cells upon CXCR3 blockade in vivo that is consistent with the mild increase of tumor clearance mediated by *Cxcr3* deficient IL-12/15/18-stimulated NK cells in the short term. This result can be associated with modulation of CXCR3 expression levels on activated cells: while IL-15 activated cells progressively increase expression of CXCR3, IL-12/15/18 activated cells rapidly downmodulate CXCR3 receptor expression thus becoming insensitive to its inhibitory effect on BM homing.

**Fig. 7:**
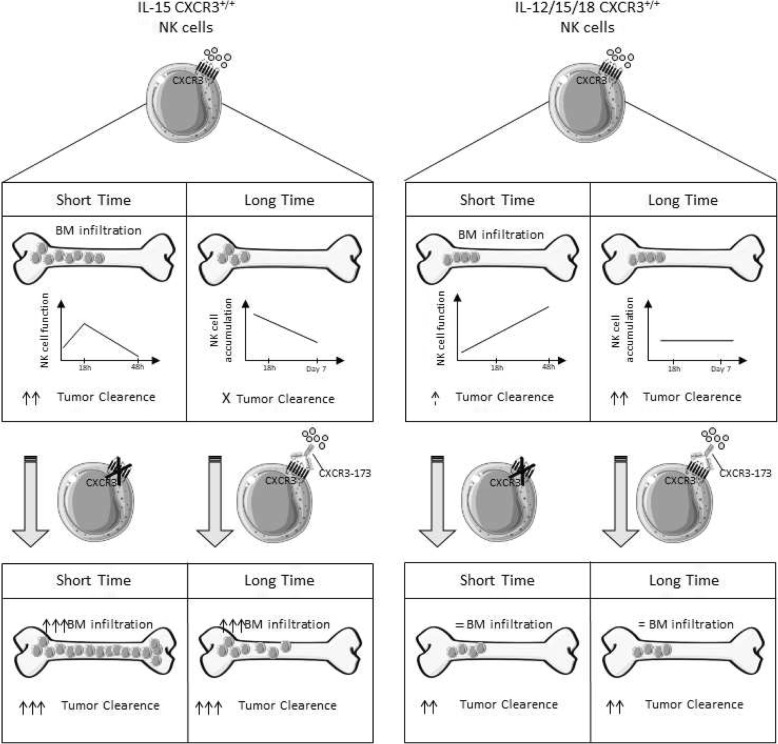
Proposed model of NK cell function in BM upon adoptive cell therapy. Upper panels: NK cells activated with IL-15 infiltrate the BM and kill tumor cells with a faster kinetics than IL12/15/18 activated cells but their effect is more transient and is thus limited to a short time frame: upon initial activation of their anti-tumor function, IL-15 activated NK cells decrease their function and number starting from 48 h after transfer and their anti-tumor effect is no more evident after 7 days; on the other hand, IL-12/15/18 activated cells are poorly effective in the short time frame, this correlating with lower infiltration and slower activation of effector function than IL-15 activated cells. Nevertheless, they persist longer than IL-15 activated cells in BM and their anti-tumor effect becomes evident at 7 days after transfer. Lower panels: CXCR3 inhibition or genetic deletion increase activated IL-15 NK cell BM infiltration, thus improving and prolonging their anti-myeloma effect up to 7 days. Higher NK cell homing corresponds to improved engraftment in BM since transferred IL-15 NK cells persist up to 7 days. Conversely, IL-12/15/18 activated cells display long-term capacity to restrain tumor growth in vivo corresponding to better persistence in BM compared to IL-15 activated cells, but their BM infiltration and anti-tumor effect is not influenced by CXCR3

Our results support the following model depicted in Fig. 7: NK cells activated with IL-15 infiltrate the BM and kill tumor cells with a faster kinetics than IL-12/15/18 activated cells but their effect is more transient and is thus limited to a short time frame. This is supported by the decreased functionality on BM IL-15 activated NK cells at 48 h after transfer and by their numeric decrease after 7 days. CXCR3 inhibition increases BM infiltration of IL-15 activated cells, thus improving and prolonging their anti-myeloma effect up to 7 days. On the other hand, IL-12/15/18 activated NK cells poorly infiltrate the BM after adoptive transfer, but their number remains stable up to 7 days and their function increases in vivo over time. Thus, they are poorly effective in the short time frame, even upon deletion of *Cxcr3* gene, but their anti-myeloma effect becomes important later after re-stimulation with IL-15.

Herein, we showed that the number of NK cells at the tumor growth site can be increased by *Cxcr3* deficiency with beneficial effect on the anti-tumor efficacy of transferred activated NK cells. Our results represent a paradigm shift in how CXCR3 regulates anti-tumor effector cell function [43]. In the context of hematological malignancy growing in BM, boosting CXCR3 function in the tumor site may be harmful to cancer immunotherapy. This is not linked to CXCR3 expression on tumor cells or immunosuppressive regulatory T or myeloid cells since CXCR3 blockade was effective only after activated NK cell transfer.

Considering current preclinical studies based on the genetic manipulation of NK cells, the genetic deletion or the silencing of CXCR3 mRNA on activated NK cells could be a useful approach to avoid the critical issue of interfering with the host immune response by mAb targeting of CXCR3 [44, 45]. Besides CXCR3 targeting, CXCL10 blockade could be a viable option in combination therapy for MM since we observed alteration of NK cell functional status in patients with high serum levels of this ligand [38]. In this regard, CXCL10 can be blocked in vivo by Eldelumab, a humanized mAb that has been used in clinical trials for rheumatoid arthritis and inflammatory bowel diseases [46].

Several pieces of evidence indicate that suppressive signals provided by MM microenvironment in BM may emerge after NK cell infiltration [47–50]. In addition, human MM develops clinical resistance to monotherapies due to clonal heterogeneity. Thus, to optimize the immunotherapeutic effect of CXCR3 targeting, combination strategies will have to deal also with the inhibition of NK cell functions promoted by factors in the tumor microenvironment.

## Conclusions

In this study we demonstrated that promotion of NK cell accumulation in BM by CXCR3 targeting is critical for the long-lasting anti-myeloma response of IL-15 activated NK cells. On the other hand, IL-12/15/18 activated cells display a longer capacity to restrain multiple myeloma growth in vivo compared to IL-15 activated cells, that seems independent of CXCR3 function due to reduced expression levels of this receptor.

In conclusion, our results support a positive effect of CXCR3 antagonism on NK cell anti-MM functions in BM. Since IL-15 is currently widely used to activate NK cells before infusion, our observations suggest that the use of IL-15-activated NK cells in combination with approaches targeting CXCR3 could be exploited to augment the therapeutic effect of current MM treatment strategies.

## Supplementary information

**Additional file 1:** Supplemental methods. (PDF)

**Additional file 2: Figure S1.** (PDF) In vivo anti-MM efficacy and in vitro functional status of activated NK cells. A) Activated (5 × 10^5^) CFSE+ NK cells obtained from splenocytes of C57BL/KaLwRij or PBS (No Cell) were i.v. transferred into MM-bearing mice 3 weeks after 5TGM1 cell injection as in Fig. 1. Tumor growth was determined by FACS analysis of intracellular IgG2b + (tumor) cells among BM cells at 48 h after transfer. B, C) Purified NK cells were activated with IL-15, IL-12/15/18 or IL-15 (10 ng/ml: used as control) for 20 h and were incubated with or without 5TGM-1 cells (E:T ratio 1:1), and with anti-NKG2D or isotype control (i.c.) mAbs. B) NK cell degranulation was assessed by FACS analysis of % CD107a + cell. Left, representative dot plots showing the frequency of CD107a^+^ on NK cells. Right, average values ± SEM of CD107a^+^ cell frequency upon 5TGM-1 and anti-NKG2D mAb stimulation subtracted of degranulation in the absence of target cells or of i.c., respectively. Degranulation of control cells with tumor: 3%; degranulation of control cells with i.c. mAb: 5%). C) Upper panels: representative histogram plot showing NKG2D expression by activated NK cells (left) and average mean fluorescence intensity (MFI) values ± SEM (right); lower panel: cytotoxic activity of activated NK cells was measured by FACS analysis upon 6 h co-incubation with CFSE+ 5TGM1 cells and staining of dead cells with 7-AAD. D) Production of IFN-γ was assessed by FACS. Left panel, representative dot plots showing the frequency of IFN-γ^+^ NK cells. Right panel, average values ± SEM of IFN-γ^+^ cell frequency upon anti-NKG2D and i.c. mAbs stimulation. IFN-γ-producing control NK cells: 3%. Student t test was performed to compare differences of IFN-γ^+^ cell frequency between cells incubated with i.c. or anti-NKG2D mAb. Results in B, C and D are representative of three independent experiments.

**Additional file 3: Figure S2.** (PDF) CXCR4 expression by *Cxcr3*^*+/+*^ and *Cxcr3*^*−/−*^ NK cells. Freshly purified, IL-15 and IL-12/15/18 activated (20 h) *Cxcr3*^*+/+*^ and *Cxcr3*^*−/−*^ NK cells were stained for CXCR4 or isotype control. Upper panels show histogram plot of overlays of CXCR4 staining in untreated and cytokine treated cells of a representative analysis. White filled histograms represent isotype control (i.c.) staining. Lower panels show average ± SEM of median fluorescence intensity (MFI) from 3 independent analysis.

**Additional file 4: Figure S3.** (PDF) Anti-MM efficacy of IL-15 activated WT versus *Cxcr3* deficient NK cells. A) Activated NK cells (5 × 10^5^) from *Cxcr3*^*+/+*^ or *Cxcr3*^*−/−*^ mice were transferred to mice two weeks after 5 T33 cell injection and tumor burden was calculated after 48 h. Graph shows the average ± SEM of frequency of tumor cells in BM and spleen from two independent experiments using a total of at least 4 animals per group. One-way ANOVA test was used to compare multiple groups. *, *P* < 0.05. B) IL-15 activated NK cells (5 × 10^5^) from *Cxcr3*^*+/+*^ or *Cxcr3*^*−/−*^ mice were transferred to MM-bearing mice as described in Fig. 4 and % of tumor cells in spleen is shown. C) IL-15 activated NK cells were transferred to mice 3 weeks after 5TGM1 cell injection. Control hamster IgG or CXCR3–173 mAb were i.v. administered one day before and the day of NK cell transfer. Donor NK cell tissue distribution was analyzed 18 h after transfer.

**Additional file 5: Figure S4.** (PDF). In vitro and in vivo expression kinetics of chemokine receptors on activated NK cells. A) Activated NK cells were labeled with 2.5 μM CFSE and adoptively transferred in mice 3 weeks after tumor cell injection following the experimental protocols depicted in Figs. 1 and 5. BM cells were isolated after 2 and 7 days and labeled with anti-CXCR4 mAb or isotype control along with anti-CD3 and anti-NK1.1. CXCR4 expression was evaluated on CFSE+ NK cells by FACS analysis. Left panels: representative histogram plots showing CXCR4 (Filled grey) expression by activated donor NK cells versus isotype control (filled white) staining. Right panels: average values ± SEM of MFI (*n* = 4 in two independent experiments). C) Purified NK cells were activated with IL-15, IL-12/15/18 for 20 h (control cells: IL-15 10 ng/ml), washed and rested in medium supplemented with IL-15 (10 ng/ml). Cells were harvested at the indicated time points and CXCR3 and CXCR4 expression was determined by FACS analysis. Results represent average values ± SEM of MFI of at least two independent analysis.

**Additional file 6: Figure S5.** (PDF). Chemokine expression in BM and tumor cells upon transfer of activated NK cells. Activated NK cells were labeled with 2.5 μM CFSE and adoptively transferred in mice 3 weeks after tumor cell injection following the experimental protocols depicted in Figs. 1 and 5. BM cells were isolated after 2 and 7 days. A) Left panels: NK cell number was determined by FACS analysis of CD3-NK1.1+ cells within donor CFSE- cells. Right panels: CXCL10 expression was determined in the bone marrow extracellular fluids from MM-bearing mice by ELISA. Histograms show mean values ± SEM of CXCL10 concentration (n = 4 from two experiments). B) Tumor cells were purified from pooled BM of tumor-bearing mice upon adoptive transfer of activated NK cells or PBS injection (no cell) and upon recombinant IL-15 administration (*n* = 3 mice group) at 2 days 7 days after adoptive transfer, respectively. CXCL10 and CXCL12 expression was measured by intracellular staining and FACS analysis (histogram plots) or by ELISA.

## Data Availability

Data sharing is not applicable to this article as no datasets were generated or analysed during the current study.

## Abbreviations

BM: Bone marrow
CFSE: Carboxyfluorescein succinimidyl ester
CXCL: Chemokine (C-X-C motif) ligand
CXCR: Chemokine (C-X-C motif)
FBS: Fetal bovine serum
IFN: Inteferon
Ig: Immunoglobulin
IL: Interleukin
MFI: Median Fluorescence Intensity
MM: multiple myeloma
NK: Natural killer
NKG2D: Natural Killer Group 2D
PBS: Phosphate buffered saline
