# Supplementary material for: Targeting of CXCR3 improves anti-myeloma efficacy of adoptively transferred activated natural killer cells
Source: J Immunother Cancer. 2019 Nov 7;7:290. doi: 10.1186/s40425-019-0751-5 (PMC6839099; doi:10.1186/s40425-019-0751-5)
Supplement: Supplementary file 1 — Additional file 1: Supplemental methods. (PDF) [file 40425_2019_751_MOESM1_ESM.pdf]

Supplemental Methods

***In vitro* functional assays**

For degranulation assays, activated NK cells were washed in RPMI 10% FBS, Hepes 10 mM and then resuspended with 5TGM1 cell line in the same medium supplemented with monensin (50 µM) and IL-2 (200 U/ml) at 1:1 effector: target (E/T) ratio. Cells were then incubated at 37°C, 5% CO_2 4 hours in 96 well-plate. During the last 2 hours, FITC-conjugated anti-mouse CD107a or IgG was added directly into each well. Afterwards, cells were harvested and analyzed by FACS. To analyse degranulation in response to NKG2D activation, cells were incubated in 96 well previously coated with anti-NKG2D mAb (CX5) or isotype Control (LTF2) (50µg/ml).

For IFN-γ production, activated NK cells were maintained in complete medium in presence of Brefeldin A (10 µg/ml) for 4 hours and then analyzed by intracellular staining using Cytofix/Cytoperm TM Fixation/Permeabilization kit following the manufacturer's instructions and FACS analysis.

To measure NK cell cytotoxicity against the 5TGM1 cells, target cells were labelled with 2 µM of CFSE. Effector cells (E) were mixed with target cells (T) at E/T ratios ranging from 8/1 to 0.125/1. After 6 h and 16 h of incubation at 37°C, cells were labeled with 7-AAD and the percentage of specific lysis was evaluated by FACS as % of 7-AAD+ inside CFSE+ cells subtracted of % of 7-AAD of CFSE+ cells incubated alone.

Chemokine measurements

BM cells were flushed from tibia and femurs to obtain single cell suspension, washed and seeded at $4x10^6$/ml in 500 µl of complete medium. Tumor cells from pooled BM samples were enriched using NK Cell Isolation Kit and plated on plastic for 3 hours to deplete adherent cells. The remaining non-adherent cells (> 85% CD138+) were collected and plated ($5x10^5$/ml) in 200 µl with (for intracellular staining) or without (for ELISA assay) addition of Brefeldin A. After 24 h, supernatants were

collected and stored at -80°C until usage, while cells were processed for intracellular staining were permeabilized as above and incubated (45 min RT) with goat anti- mouse CXCL10 (AF466-NA and CXCL12 (AF-310-NA) followed by 20 min incubation with rat anti-goat IgG (Jackson immunoresearch). Chemokine concentration in supernatant was determined using specific DuoSet Kits (R&D, Minneapolis, MI) according to manufacturer's instruction.
