# Supplementary figures and images for: Targeting of CXCR3 improves anti-myeloma efficacy of adoptively transferred activated natural killer cells
Source: J Immunother Cancer. 2019 Nov 7;7:290. doi: 10.1186/s40425-019-0751-5 (PMC6839099; doi:10.1186/s40425-019-0751-5)

Figure S1

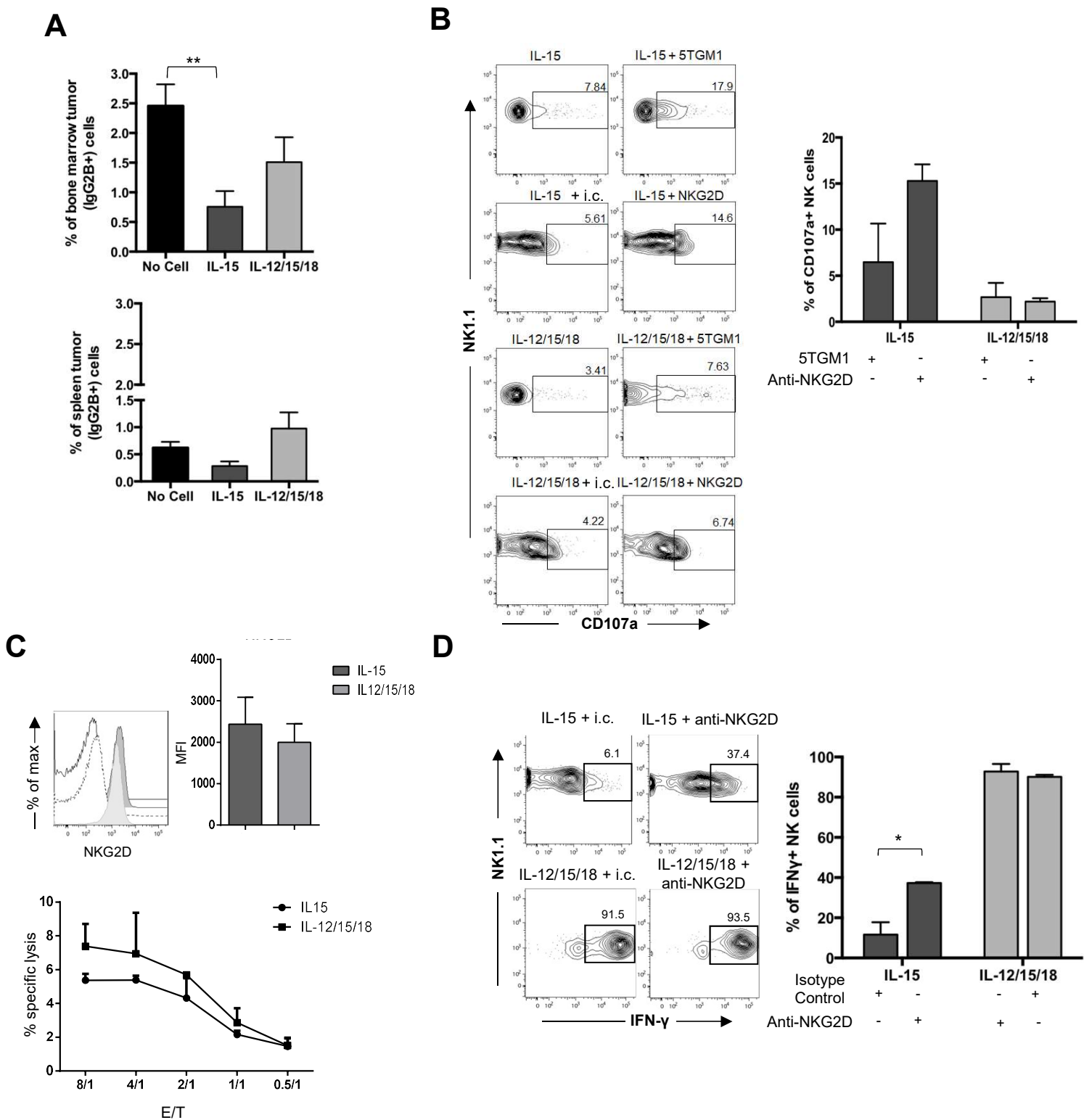

Supplement: Supplementary file 2 — Additional file 2: Figure S1. (PDF) In vivo anti-MM efficacy and in vitro functional status of activated NK cells. A) Activated (5 × 10^5^) CFSE+ NK cells obtained from splenocytes of C57BL/KaLwRij or PBS (No Cell) were i.v. transferred into MM-bearing mice 3 weeks after 5TGM1 cell injection as in Fig. 1. Tumor growth was determined by FACS analysis of intracellular IgG2b + (tumor) cells among BM cells at 48 h after transfer. B, C) Purified NK cells were activated with IL-15, IL-12/15/18 or IL-15 (10 ng/ml: used as control) for 20 h and were incubated with or without 5TGM-1 cells (E:T ratio 1:1), and with anti-NKG2D or isotype control (i.c.) mAbs. B) NK cell degranulation was assessed by FACS analysis of % CD107a + cell. Left, representative dot plots showing the frequency of CD107a+ on NK cells. Right, average values ± SEM of CD107a+ cell frequency upon 5TGM-1 and anti-NKG2D mAb stimulation subtracted of degranulation in the absence of target cells or of i.c., respectively. Degranulation of control cells with tumor: 3%; degranulation of control cells with i.c. mAb: 5%). C) Upper panels: representative histogram plot showing NKG2D expression by activated NK cells (left) and average mean fluorescence intensity (MFI) values ± SEM (right); lower panel: cytotoxic activity of activated NK cells was measured by FACS analysis upon 6 h co-incubation with CFSE+ 5TGM1 cells and staining of dead cells with 7-AAD. D) Production of IFN-γ was assessed by FACS. Left panel, representative dot plots showing the frequency of IFN-γ+ NK cells. Right panel, average values ± SEM of IFN-γ+ cell frequency upon anti-NKG2D and i.c. mAbs stimulation. IFN-γ-producing control NK cells: 3%. Student t test was performed to compare differences of IFN-γ+ cell frequency between cells incubated with i.c. or anti-NKG2D mAb. Results in B, C and D are representative of three independent experiments. [file 40425_2019_751_MOESM2_ESM.pdf]

Figure S2

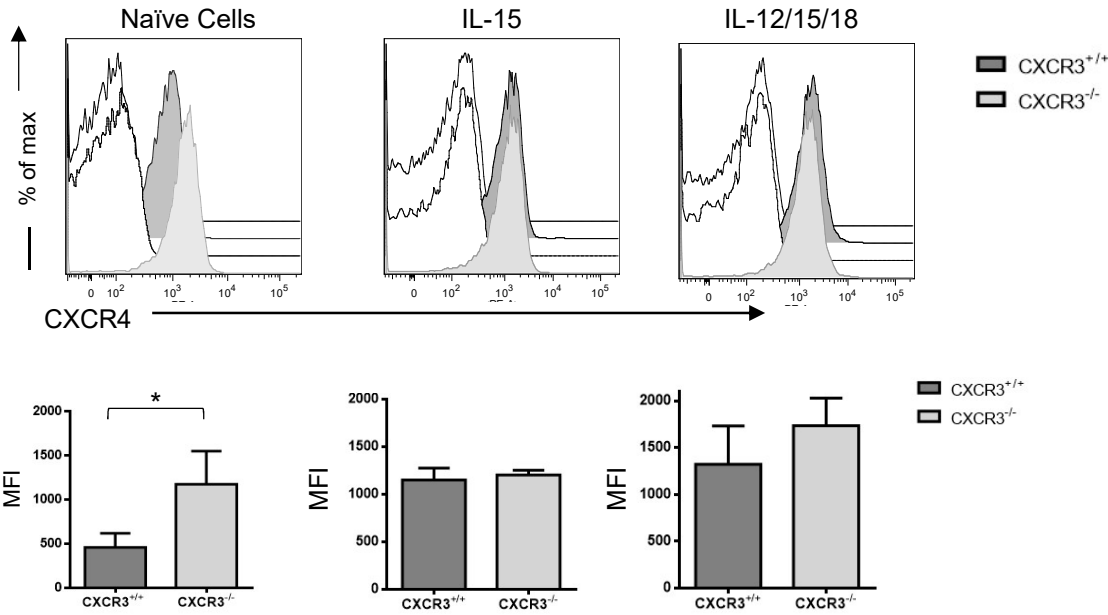

Supplement: Supplementary file 3 — Additional file 3: Figure S2. (PDF) CXCR4 expression by Cxcr3+/+ and Cxcr3−/− NK cells. Freshly purified, IL-15 and IL-12/15/18 activated (20 h) Cxcr3+/+ and Cxcr3−/− NK cells were stained for CXCR4 or isotype control. Upper panels show histogram plot of overlays of CXCR4 staining in untreated and cytokine treated cells of a representative analysis. White filled histograms represent isotype control (i.c.) staining. Lower panels show average ± SEM of median fluorescence intensity (MFI) from 3 independent analysis. [file 40425_2019_751_MOESM3_ESM.pdf]

Figure S3

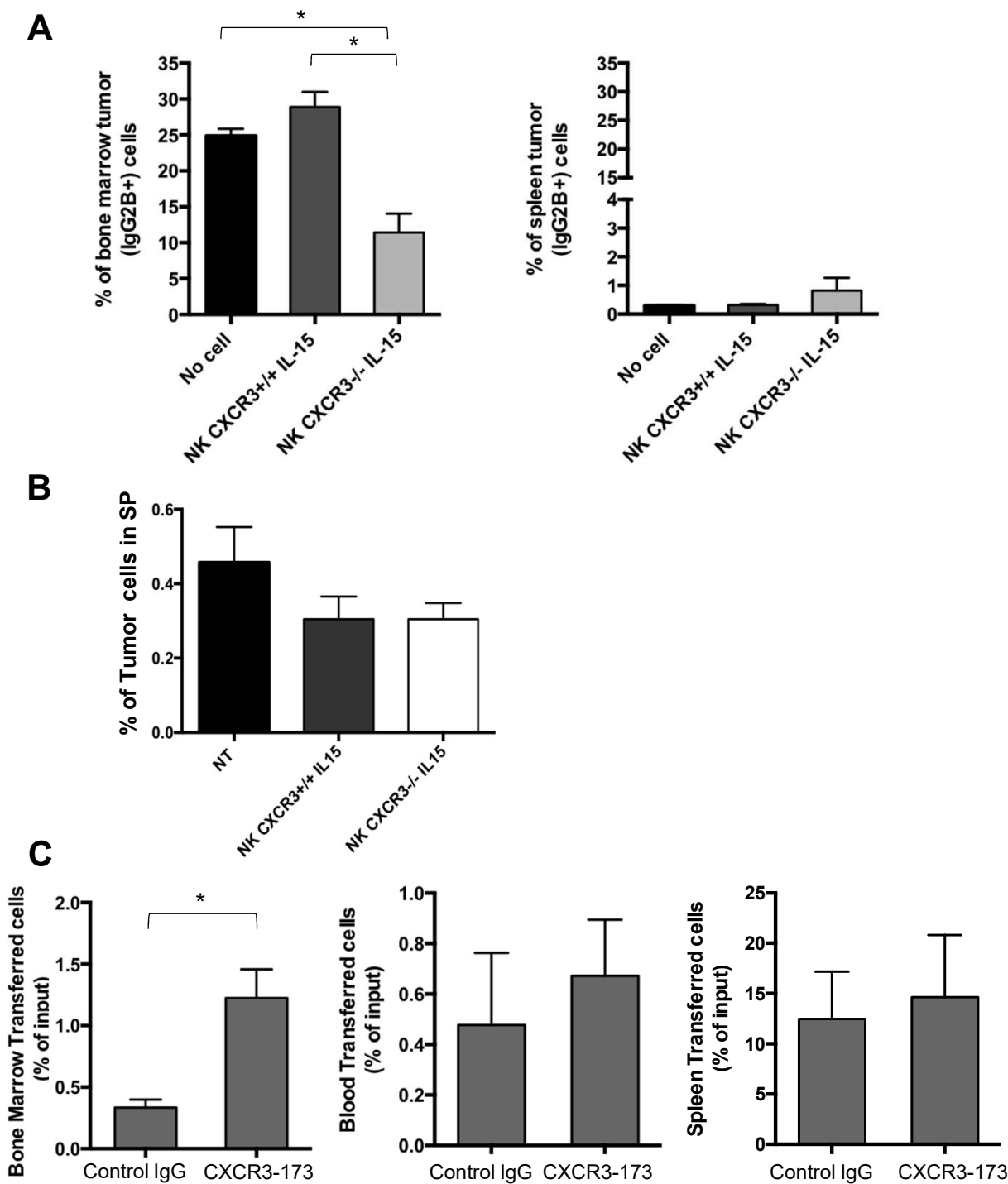

Supplement: Supplementary file 4 — Additional file 4: Figure S3. (PDF) Anti-MM efficacy of IL-15 activated WT versus Cxcr3 deficient NK cells. A) Activated NK cells (5 × 10^5^) from Cxcr3+/+ or Cxcr3−/− mice were transferred to mice two weeks after 5 T33 cell injection and tumor burden was calculated after 48 h. Graph shows the average ± SEM of frequency of tumor cells in BM and spleen from two independent experiments using a total of at least 4 animals per group. One-way ANOVA test was used to compare multiple groups. *, P < 0.05. B) IL-15 activated NK cells (5 × 10^5^) from Cxcr3+/+ or Cxcr3−/− mice were transferred to MM-bearing mice as described in Fig. 4 and % of tumor cells in spleen is shown. C) IL-15 activated NK cells were transferred to mice 3 weeks after 5TGM1 cell injection. Control hamster IgG or CXCR3–173 mAb were i.v. administered one day before and the day of NK cell transfer. Donor NK cell tissue distribution was analyzed 18 h after transfer. [file 40425_2019_751_MOESM4_ESM.pdf]

Figure S4

A

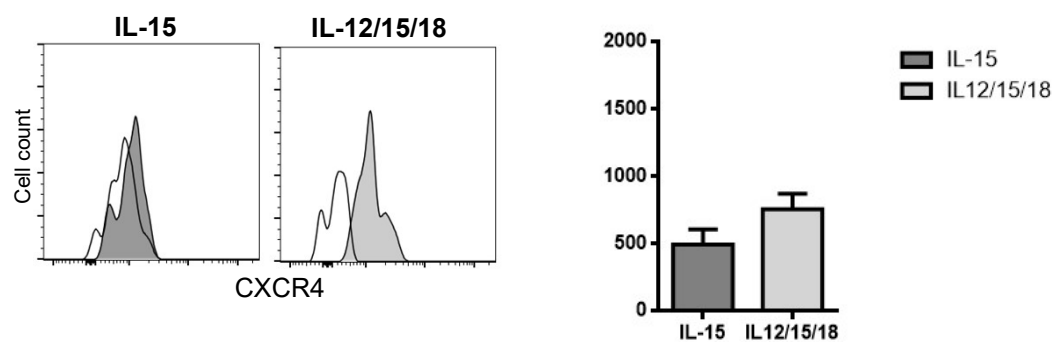

B

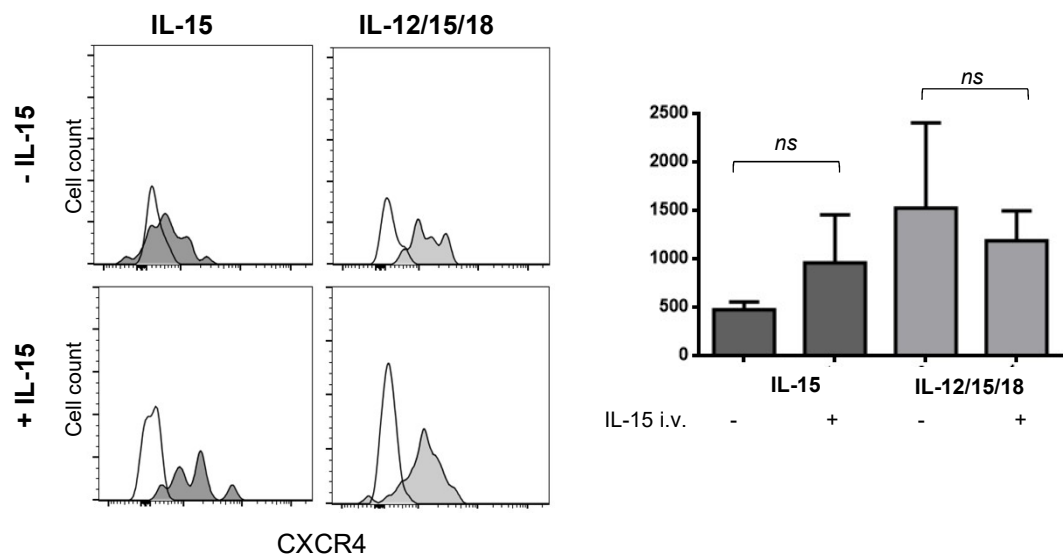

C

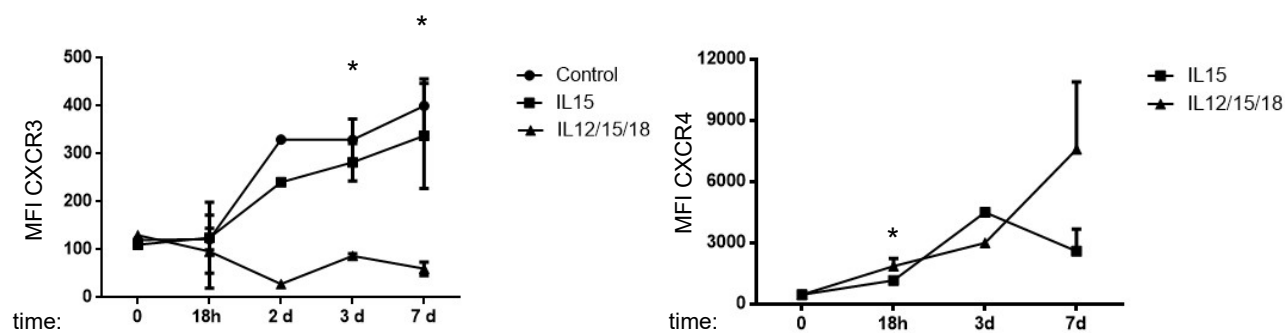

Supplement: Supplementary file 5 — Additional file 5: Figure S4. (PDF). In vitro and in vivo expression kinetics of chemokine receptors on activated NK cells. A) Activated NK cells were labeled with 2.5 μM CFSE and adoptively transferred in mice 3 weeks after tumor cell injection following the experimental protocols depicted in Figs. 1 and 5. BM cells were isolated after 2 and 7 days and labeled with anti-CXCR4 mAb or isotype control along with anti-CD3 and anti-NK1.1. CXCR4 expression was evaluated on CFSE+ NK cells by FACS analysis. Left panels: representative histogram plots showing CXCR4 (Filled grey) expression by activated donor NK cells versus isotype control (filled white) staining. Right panels: average values ± SEM of MFI (n = 4 in two independent experiments). C) Purified NK cells were activated with IL-15, IL-12/15/18 for 20 h (control cells: IL-15 10 ng/ml), washed and rested in medium supplemented with IL-15 (10 ng/ml). Cells were harvested at the indicated time points and CXCR3 and CXCR4 expression was determined by FACS analysis. Results represent average values ± SEM of MFI of at least two independent analysis. [file 40425_2019_751_MOESM5_ESM.pdf]

Figure S5

A

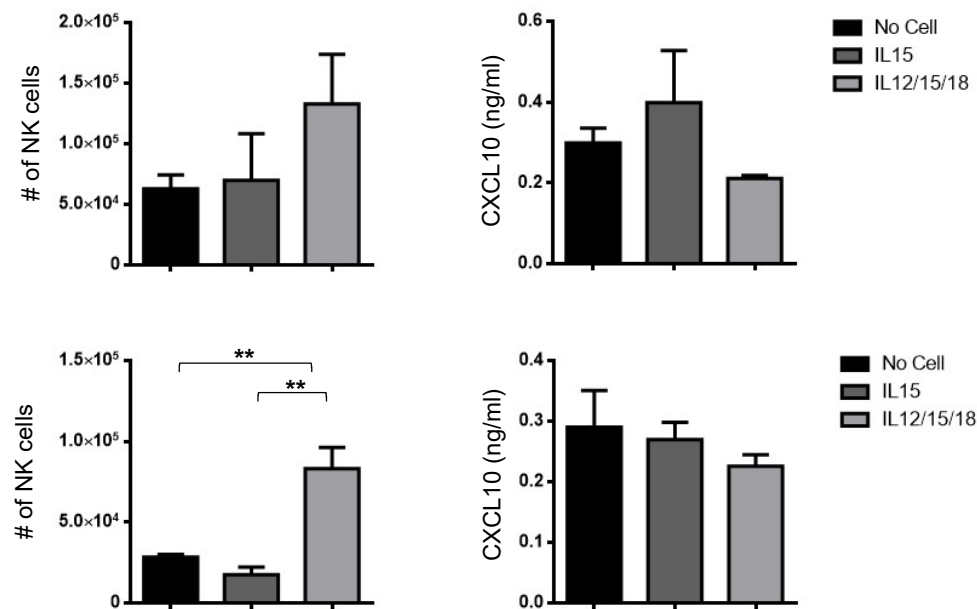

B

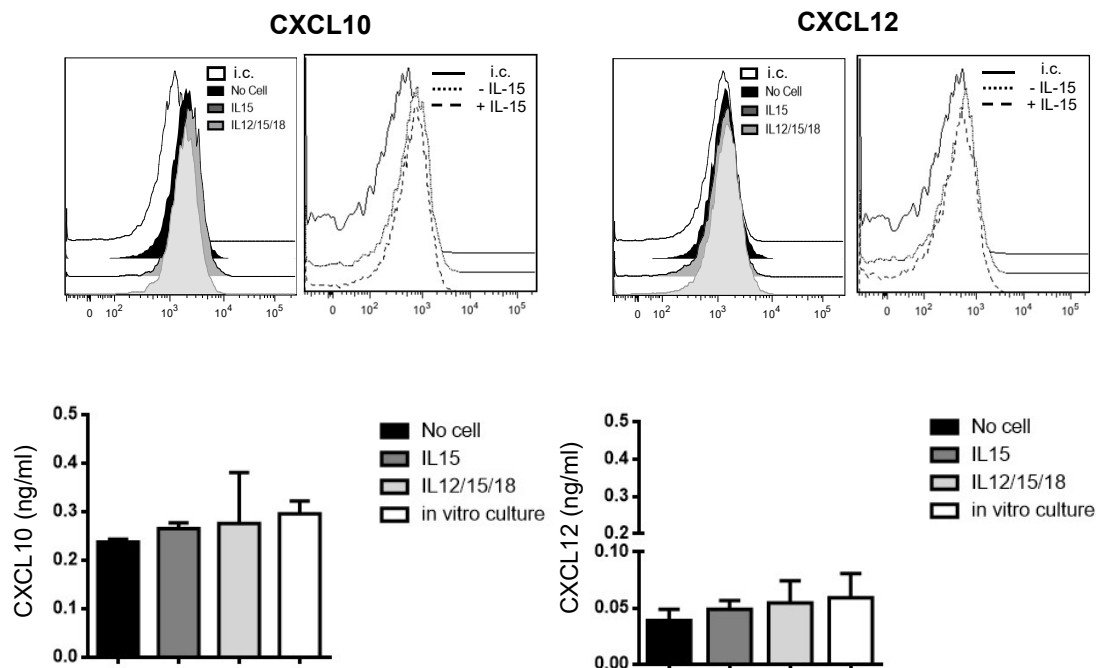

Supplement: Supplementary file 6 — Additional file 6: Figure S5. (PDF). Chemokine expression in BM and tumor cells upon transfer of activated NK cells. Activated NK cells were labeled with 2.5 μM CFSE and adoptively transferred in mice 3 weeks after tumor cell injection following the experimental protocols depicted in Figs. 1 and 5. BM cells were isolated after 2 and 7 days. A) Left panels: NK cell number was determined by FACS analysis of CD3-NK1.1+ cells within donor CFSE- cells. Right panels: CXCL10 expression was determined in the bone marrow extracellular fluids from MM-bearing mice by ELISA. Histograms show mean values ± SEM of CXCL10 concentration (n = 4 from two experiments). B) Tumor cells were purified from pooled BM of tumor-bearing mice upon adoptive transfer of activated NK cells or PBS injection (no cell) and upon recombinant IL-15 administration (n = 3 mice group) at 2 days 7 days after adoptive transfer, respectively. CXCL10 and CXCL12 expression was measured by intracellular staining and FACS analysis (histogram plots) or by ELISA. [file 40425_2019_751_MOESM6_ESM.pdf]
